# Activation of Adenosine Monophosphate-Activated Protein Kinase Drives the Aerobic Glycolysis in Hippocampus for Delaying Cognitive Decline Following Electroacupuncture Treatment in APP/PS1 Mice

**DOI:** 10.3389/fncel.2021.774569

**Published:** 2021-11-16

**Authors:** Jianhong Li, Bingxue Zhang, Weiwei Jia, Minguang Yang, Yuhao Zhang, Jiayong Zhang, Le Li, Tingting Jin, Zhifu Wang, Jing Tao, Lidian Chen, Shengxiang Liang, Weilin Liu

**Affiliations:** ^1^TCM Rehabilitation Research Center of SATCM, Fujian University of Traditional Chinese Medicine, Fuzhou, China; ^2^National-Local Joint Engineering Research Center of Rehabilitation Medicine Technology, Fujian University of Traditional Chinese Medicine, Fuzhou, China; ^3^College of Rehabilitation Medicine, Fujian University of Traditional Chinese Medicine, Fuzhou, China

**Keywords:** electroacupuncture, Alzheimer’s disease, aerobic glycolysis, learning and memory, adenosine monophosphate-activated protein kinase (AMPK)

## Abstract

Aerobic glycolysis (AG), an important pathway of glucose metabolism, is dramatically declined in Alzheimer’s disease (AD). AMP-activated protein kinase (AMPK) is a key regulator to maintain the stability of energy metabolism by promoting the process of AG and regulating glucose metabolism. Interestingly, it has been previously reported that electroacupuncture (EA) treatment can improve cognitive function in AD through the enhancement of glucose metabolism. In this study, we generated AMPK-knockdown mice to confirm the EA effect on AMPK activation and further clarify the mechanism of EA in regulating energy metabolism and improving cognitive function in APP/PS1 mice. The behavioral results showed that EA treatment can improve the learning and memory abilities in APP/PS1 mice. At the same time, the glucose metabolism in the hippocampus was increased detected by MRI-chemical exchange saturation transfer (MRI-CEST). The expression of proteins associated with AG in the hippocampus was increased simultaneously, including hexokinase II (HK2), 6-phosphofructo-2-kinase/fructose-2,6-biphosphatase 3 (PFKFB3), and pyruvate kinase M2 (PKM2). Moreover, the knockdown of AMPK attenuated AG activated by EA treatment. In conclusion, this study proves that EA can activate AMPK to enhance the process of AG in the early stage of AD.

## Introduction

Alzheimer’s disease (AD) is the most common neurodegenerative disease, which is clinically characterized by progressive cognitive impairment, accounting for 60–80% of all dementia cases. According to the AD report of 2018, approximately 50 million people worldwide are affected by dementia, with the number expected to reach 152 million by 2050 (Peprah and McCormack, 2019). Glucose metabolism dysfunction is a serious manifestation of AD, which even emerges earlier than cognitive decline and dementia (Daulatzai, 2017; Kuehn, 2020). Fluorodeoxyglucose positron emission tomography (FDG-PET) could measure glucose metabolism within the brain, which has been used to aid dementia diagnosis (Arbizu et al., 2018; Wilson et al., 2019). Research in this area has shown that the glucose metabolism was impaired in several brain regions during AD progression, and this dysfunction usually starts in the memory-related brain regions, such as the entorhinal cortex, parietal lobe, posterior cingulate cortex, notably the hippocampus and associated temporal areas (Minoshima et al., 1997; Mosconi, 2005). Some researchers pointed out that many pathological changes that arise downstream of glucose metabolism dysfunction may be the cause of neuronal degenerative changes in AD (Chen and Zhong, 2013).

The glucose metabolism route consists of glycolysis and mitochondrial oxidative phosphorylation, and the latter is the main pathway for energy production in mitochondrion promoting the survival and function of neurons (Akram, 2014; Jin and Diano, 2018). In the past century, Otto Warburg first observed that aerobic glycolysis (AG) was utilized to support cancer cell proliferation (Riester et al., 2018). Since then, AG has been a major focus in the field of cancer metabolic research. In recent years, several studies have found that abnormal AG is associated with AD. As the review of the literature concluded that glucose metabolism dysfunction during AD progression, including decreased glucose uptake and glucose utilization, may be related to the impairment of AG, leading to permanent neurological impairments (An et al., 2018). In the initial stage of AD, amyloid-beta (Aβ) fragments could cause abnormal mitochondrial dynamics, resulting in mitochondrial dysfunction and neuronal damage, which makes AG an important energy supplement way (Manczak et al., 2016; Yin et al., 2020). Another study has found that the AG was increased in response to the inhibition of oxidative phosphorylation in cultured cortical neurons (Santangelo et al., 2021). Moreover, some scholars have found that the process of AG could attenuate neuronal death caused by Aβ oligomers (Newington et al., 2011). Further studies found that AG can improve the ability of neurons to attenuate Aβ-mediated neurotoxicity by increasing the expression of pyruvate dehydrogenase kinase 1 (PDK1) and lactate dehydrogenase A (LDHA) (Newington et al., 2012).

Interestingly, animal studies have found that electroacupuncture (EA) treatment could elevate glucose metabolism (Dong et al., 2015; Liu et al., 2017). A large number of studies have shown that EA is a proven means of therapeutic intervention to improve cognitive function and life quality of patients with AD (Zhou et al., 2015; Jia et al., 2017). In the past studies, we have found that EA at the DU20 and the DU24 acupoints could improve the cognitive performance of APP/PS1 mice through increasing glucose metabolism (Lin et al., 2016; Liu et al., 2017). However, the mechanism of EA treatment improving cognitive function in AD is needed for further exploration. Studies have demonstrated that the reduction of AMP-activated protein kinase (AMPK) could result in progressive loss of neuronal functions, suggesting that restoration of AMPK expression may serve as a potential therapeutic target for AD (Yang et al., 2015). AMPK is considered a key regulator of energy metabolism as it can control glucose metabolism and regulate glycolysis flux (Yan et al., 2018). Studies have indicated that AMPK could promote glucose metabolism by regulating proteins associated with AG, such as hexokinase II (HK2), 6-phosphofructo-2-kinase/fructose-2,6-biphosphatase 3 (PFKFB3), and pyruvate kinase M2 (PKM2) (Liu et al., 2019; Xu et al., 2019). Additionally, studies have also found that the expression levels of HK2, PFKFB3, and PKM2 were changed in AD, which may be associated with the impairment of memory and other cognitive function (Bigl et al., 1999; Cisternas et al., 2019). Taking into account these findings, we hypothesized that EA treatment might enhance AG through AMPK to improve the learning and memory abilities in APP/PS1 mice, and in this study, AMPK-knockdown mice were generated to confirm this hypothesis.

## Materials and Methods

### Animals and Ethics

In this study, APP/PS1 double-transgenic mice were used as the AD animal model. APP/PS1 double-transgenic mice [B6C3-TG (APPswe, PSEN1dE9) 85Dbo/MmJNju], Camk2a-cre mice, and wild type (WT) mice were obtained from Nanjing University – Nanjing Biomedical Research Institute; AMPKa1(loxp/loxp) mice were purchased from Xiamen University (Stock No: 014141). The animals were fed in the SPF animal experimental center of Fujian University of Traditional Chinese Medicine, each cage for 4–5 animals, free to water and food. The SPF room was set up for a standard day and night system (12/12 h light/dark cycle), with the temperature at 22° and humidity at 50–70%. APP/PS1 mice applied in this experiment were obtained by crossing male APP/PS1 mice to female WT mice. To produce AMPK-knockdown APP/PS1 mice, AMPKa1(loxp/loxp) mice were interbred with APP/PS1 mice to obtain APP/PS1 + AMPKa1(loxp/loxp) mice, and these mice were mated to Camk2a-cre mice to obtain APP/PS1 + AMPKa1^(+/–)^ mice. The genotypes of the offsprings were identified using the polymerase chain reaction. All experiments involving animals were approved by the Fujian University of Traditional Chinese Medicine Animal Experimentation Ethics Committee, and the experiments are operated and implemented in strict accordance with the provisions of the national animal protection laws and regulations.

### Experimental Protocol

**FIGURE 1:**
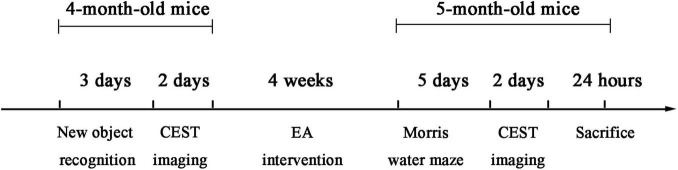
The detailed timeline of the experiments, including the electroacupuncture (EA) treatment and time sequence.

After gene identification, 4-month-old mice were divided into five groups as follows: WT group, AD group, AD + EA group, AD + AMPK^(+/–)^ group, and AD + AMPK^(+/–)^ + EA group, each group with 10 mice. The detailed experimental timeline is depicted in Figure 1. For EA treatment, the mice were fixed on a special device. Mice were sedated by lightly stroking the body. Stainless steel acupuncture steel (0.32 mm diameter; Huatuo, Suzhou Medical Appliance Factory, Suzhou, China) was inserted to a depth of 2–3 mm at DU20 and DU24 acupoints according to the acupuncture point map of experimental animals and the acupuncture and moxibustion science. Then, the EA instrument (model: G6805; Suzhou Medical Appliance Factory, Suzhou, China) was used with a frequency of sparse and dense waves of 1/20 Hz, a voltage of 2 V, for 30 min, once a day, 5 times a week, for 4 weeks.

### Novel Object Recognition Test

The novel object recognition test was used to assess recognition memory. It was performed according to the previously explained method (Bevins and Besheer, 2006). In brief, the apparatus includes a rectangular open-field box, object “A,” object “a,” and object “B,” in which the object “A” is the same as object “a.” One day before the formal experiment, the mice were put into the open-field box for 10 min for acclimation. In the training session, two identical objects “A” and “a” were placed on the two adjacent corners of the open-field box, and then the mice were put into the open-field box to explore for 10 min. Notably, 1 and 24 h after the training session, the mice were reintroduced to the same task, but one of the familiar objects applied during the training session was replaced by a novel object “B,” and the mice were put into the open-field box to explore for 5 min. During the experiment, the trajectory and behavior including the time of each mouse contact with the objects were recorded using the camera of Super Maze. After each trial, the open-field box and objects were cleaned with 70% of ethanol to eliminate the presence of any olfactory cues. Evaluation index: the mice were considered to be exploring when the animal nose was toward the object within 2 cm. In this study, recognition index = {Time(B)/[Time(A) + Time(B)]}.

### Morris Water Maze Test

Morris water maze test was used to evaluate the spatial learning and memory abilities of mice after EA intervention. A cylindrical tank with a radius of 60 cm surrounded by a wall of 50 cm high and filled with opaque water was used. The depth of the water is 30 cm, and the temperature is maintained at 25 ± 2°C. A transparent circular platform with a diameter of 6 cm was hidden below the water surface. Four spatial cues with different shapes and colors were pasted above the edge of the tank. The mice were allowed 16 sessions of a swim on the first 4 days, 4 sessions each day. In this stage, the mice were put into the water from four different quadrants, and the time spent to reach the escape platform was measured as escape latency. If the mice climbed to the escape platform within 90 s, record the time as escape latency, and if the mice did not reach the escape platform, record the escape latency as 90 s, and the mice were guided to the platform by the experimenter to learn for 15 s. On the 5th day, the escape platform in the Morris water maze was removed, and the mice were directly put into the opposite quadrant where the original platform was located to swim for 90 s. The swimming trajectory and behavior including the escape platform crossing times and the time spent in the target quadrant were tracked using the SuperMaze small animal behavior video analysis system.^1^

### MRI-Chemical Exchange Saturation Transfer Imaging

The MRI-chemical exchange saturation transfer (MRI-CEST) scans in the 7.0 T magnetic resonance imaging small animal system (Bruker Biospec, Ettlingen, Germany) was applied to detect the levels of glucose metabolism, each group for four mice. One day before CEST imaging, food was removed, and the mice were fasted 12 h with free water access. Mice were anesthetized with 1.5% isoflurane. A volume coil with a radius of 3.6 cm was used as the transmitter and an orthogonal single-channel surface coil as the receiver during scanning. The T2WI image is scanned for reference prior to CEST imaging. The MAPSHIM program was utilized to calculate the first- and second-order parameters to optimize B0 field homogeneity.

The detailed acquisition parameters for T2WI were as follows: repetition time (TR) = 5,000 ms, echo time (TE) = 105.02 ms, field of view (FOV) = 16 mm × 16 mm, image matrix = 256 × 256, and slice thickness = 1 mm. CEST consisted of a continuous pulse for the 43 offset frequencies from –1,800 to 1,800 Hz with the interval of 90 Hz. The detailed acquisition parameters were as follows: TR = 5,000 ms, TE = 35.45 ms, B1 = 3.7 μT, FOV = 16 mm × 16 mm, image matrix = 45 × 45, and slice thickness = 1 mm.

Using the asymmetric magnetization transfer ratio (MTRasym), asymmetric equation: MTRasym (Δ*ω*) = [Ssat (–Δ*ω*)–Ssat (Δ*ω*)]/S0 (van Zijl and Yadav, 2011), and calculated CEST glucose metabolism image, including Δ*ω*, the exchange of content and free water frequency difference, the free water saturation before signal as S0, and free water for the Ssat signal after saturation were determined. The voxel counts were normalized by the mean intensity of the brain in the metabolism image of each mice. Then, ITK-snap^2^ was also used to determine the left and right hippocampus regions of interest (ROI) manually and extract glucose metabolism values for each mouse.

### Western Blotting

The hippocampus was extracted for Western blotting, four mice in each group. The RIPA lysis buffer and the BCA Kit were used to extract total protein and determine protein concentration, respectively. A total of 50 μg proteins were resolved by 10% sodium dodecyl-sulfate-polyacrylamide gel electrophoresis (SDS-PAGE) and then transferred to activated polyvinylidene fluoride (PVDF) membranes. The membranes were blocked with 5% skimmed milk for 2 h at room temperature, incubated with antibodies against AMPK (cat. No. ab131512, 1:1,000), PFKFB3 (cat. No. 13763-1-AP, 1:3,000), HK2 (cat. No. 22029-1-AP, 1:5,000), and PKM2 (cat. No. Ab137852, 1:1,000) at 4°C overnight, and subsequently incubated with horseradish peroxidase-conjugated (HRP-conjugated) secondary antibody (1:8,000) for 2 h. The protein bands were visualized with enhanced chemiluminescence and imaged with the Bio-Image Analysis system (Bio-Rad Laboratories, Inc.).

### Statistical Analysis

The statistical differences were evaluated using SPSS version 24.0 (Released 2016, IBM SPSS Statistics for Windows, IBM Corp., Armonk, NY). The values are expressed as means ± SEM. The escape latency of the Morris water maze was analyzed using the repeated measurement analysis method, other results were analyzed by one-way ANOVA, and multiple comparisons were performed using the Fisher’s least significant difference (LSD) or Games-Howell test. The difference was considered to be significant when *P* < 0.05.

## Results

### Electroacupuncture Treatment Alleviated Learning and Memory Impairment of APP/PS1 Mice

**FIGURE 2:**
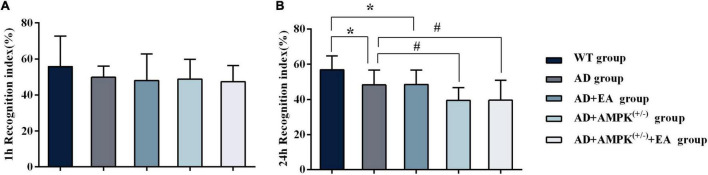
Novel object recognition test showing decreased recognition index for the novel object in 4-month-old APP/PS1 mice. **(A)** The 1 h recognition index of five groups before EA intervention; **(B)** The 24 h recognition index of five groups before EA intervention. *P < 0.05 vs. the wild type (WT) group; ^#^*P* < 0.05 vs. the Alzheimer’s disease (AD) group.

**FIGURE 3:**
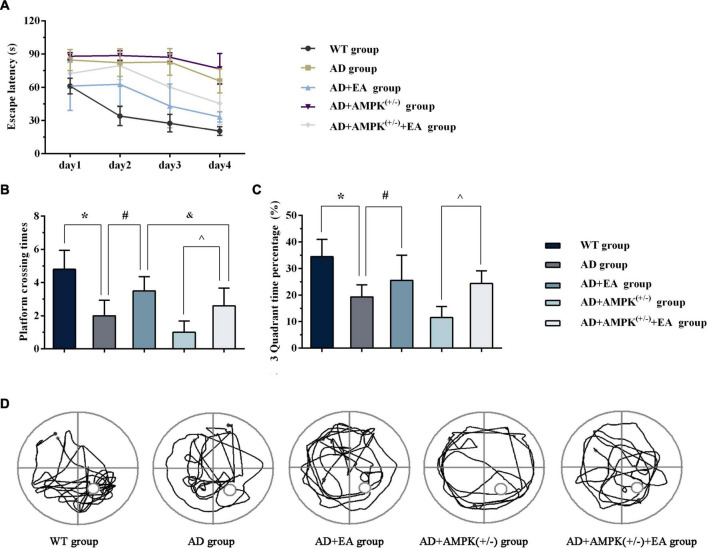
Electroacupuncture intervention could improve the learning and memory abilities of APP/PS1 mice tested by Morris water maze. **(A)** The escape latency of five groups in Morris water maze after EA intervention; **(B)** The escape platform crossing times of five groups in Morris water maze after EA intervention; **(C)** The 3rd quadrant time percentage of five groups in Morris water maze after EA intervention; **(D)** Representative images of Morris water maze in different groups. *P < 0.05 vs. the WT group; ^#^*P* < 0.05 vs. the AD group; ^∧^*P* < 0.05 vs. the AD + AMP-activated protein kinase (AMPK)^(+/–)^ group; ^&^*P* < 0.05 vs. the AD + EA group.

To observe the effects of EA treatment on the learning and memory abilities of APP/PS1 transgenic mice, the new object recognition test was applied before EA intervention in order to detect the recognition memory of each group, and the results were shown in Figure 2. The 24-h novel object recognition index of the AD group was decreased compared with the WT group (*P* < 0.05), and further analysis showed that the AD + AMPK^(+/–)^ group was decreased compared with the AD group (*P* < 0.05) (Figure 2B). However, there was no significant difference in the 1-h novel object recognition index between individual groups (Figure 2A). After EA intervention, the learning and memory abilities of each group were detected using the Morris water maze, and the results were exhibited in Figure 3. Escape latency in the AD group was increased compared with the WT group. However, escape latency in the AD + EA group was decreased compared with the AD group, and the AD + AMPK^(+/–)^ + EA group decreased compared with the AD + AMPK^(+/–)^ group (Figure 3A). The escape platform crossing times and the 3rd quadrant time proportion in the AD group were decreased compared with the WT group (*P* < 0.05); the AD + EA group increased compared with the AD group (*P* < 0.05); the AD + AMPK^(+/–)^ + EA group increased compared with the AD + AMPK^(+/–)^ group (*P* < 0.05) (Figures 3B,C). The representative images of the Morris water maze in different groups were shown in Figure 3D. These results suggested that EA at DU20 and DU24 acupoints could improve the learning and memory abilities of APP/PS1 mice.

### Electroacupuncture Treatment Increased Glucose Metabolism in Hippocampus of APP/PS1 Mice

**FIGURE 4:**
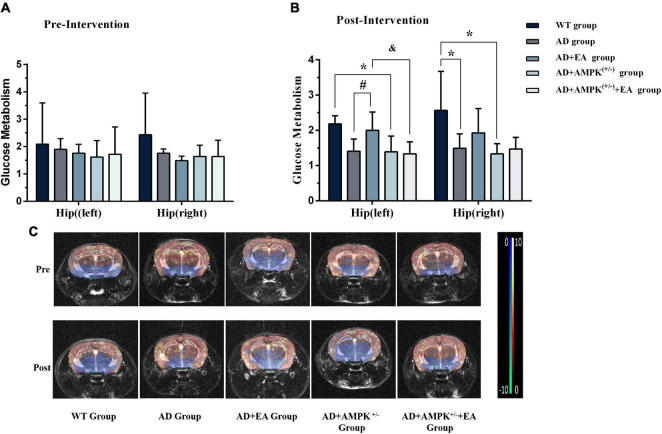
Electroacupuncture intervention could increase glucose metabolism in the hippocampus of APP/PS1 mice. **(A)** The glucose metabolism in the hippocampus of five groups before EA intervention; **(B)** The glucose metabolism in the hippocampus of five groups after EA intervention; **(C)** The typical image of glucose metabolism in the hippocampus. *P < 0.05 vs. the WT group; ^#^*P* < 0.05 vs. the AD group; ^&^*P* < 0.05 vs. the AD + EA group.

To observe the effects of EA on hippocampus glucose metabolism of APP/PS1 transgenic mice, MRI-CEST imaging was conducted before and after the EA intervention. The results before the EA intervention were shown in Figure 4A. One-way ANOVA results showed that there were no significant differences in glucose metabolism on the hippocampus (both left and right) between individual groups (*P* > 0.05). After EA intervention, the results in Figure 4B showed that the glucose metabolism in the right hippocampus of the AD group was decreased compared with the WT group (*P* < 0.05); the glucose metabolism in the hippocampus (left and right) of the AD + AMPK^(+/–)^ group were decreased compared with the WT group (*P* < 0.05 and *P* < 0.01). However, the glucose metabolism in the left hippocampus of the AD + EA group was increased compared with the AD group (*P* < 0.05). The glucose metabolism in the left hippocampus of the AD + AMPK^(+/–)^ + EA group was decreased compared with the AD + EA group (*P* < 0.05). The typical CEST image was shown in Figure 4C. It suggested that EA at DU20 and DU24 acupoints could improve the hippocampus glucose metabolism of APP/PS1 mice.

### Electroacupuncture Treatment Increased the Expression of Proteins Associated With Aerobic Glycolysis of APP/PS1 Mice

**FIGURE 5:**
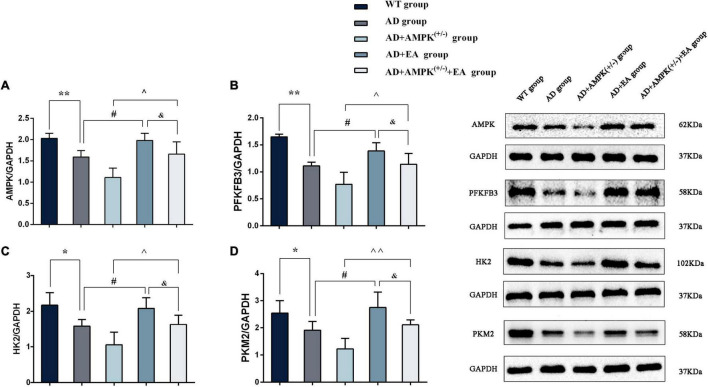
Electroacupuncture intervention could promote the expression of aerobic glycolysis (AG)-related proteins. **(A–D)** The expression of AMPK, 2,6-fructose-biphosphatase 3 (PFKFB3), hexokinase II (HK2), and pyruvate kinase M2 (PKM2) in the hippocampus of five groups after EA intervention; (E) representative protein images of each AG-related protein in different groups. *P < 0.05, **P < 0.01 vs. the WT group; ^#^*P* < 0.05 vs. the AD group; ^∧^*P* < 0.05; ^∧∧^*P* < 0.01 vs. the AD + AMPK^(+/–)^ group; ^&^*P* < 0.05 vs. the AD + EA group.

To observe the effects of EA on hippocampus AMPK and AG rate-limiting enzyme expression in APP/PS1 transgenic mice, Western blotting was performed and the results were shown in Figure 5. Compared with the WT group, the expression levels of AMPK, PFKFB3, HK2, and PKM2 were significantly decreased in the AD group (*P* < 0.01 or *P* < 0.05); compared with the AD group, the expression levels of AMPK, PFKFB3, HK2, and PKM2 were increased in the AD + EA group (*P* < 0.05); compared with the AD + AMPK^(+/–)^ group, the expression levels of AMPK, PFKFB3, HK2, and PKM2 were increased in the AD + AMPK^(+/–)^ + EA group (*P* < 0.01 or *P* < 0.05). Compared with the AD + EA group, the expression levels of AMPK, PFKFB3, HK2, and PKM2 were decreased in the AD + AMPK^(+/–)^ + EA group (*P* < 0.05). The above results showed that the AMPK activity and the AG-related proteins were decreased in the hippocampus of APP/PS1 and APP/PS1 + AMPK^(+/–)^ mice, while EA at DU20 and DU24 acupoints could activate AMPK to improve the expression levels of PFKFB3, HK2, and PKM2 in APP/PS1 transgenic mice.

## Discussion

Our previous studies have found that EA treatment could effectively elevate glucose metabolism to improve the learning and memory abilities in APP/PS1 mice (Liu et al., 2017). This study was designed to examine the underlying molecular mechanism *via* which EA treatment improved glucose metabolism in APP/PS1 mice. To elucidate this issue, the CRE-LOXP conditional knockout technology was used to generate APP/PS1 mice with AMPK knockdown. Combined with MRI-CEST imaging analysis, we confirmed that EA at the DU20 and DU24 acupoints could improve glucose metabolism in APP/PS1 mice to enhance learning and memory abilities. To gain further insight, we analyzed the proteins associated with AG in the hippocampus using the immunoblot analysis, and the results showed that the expression levels of AMPK, HK2, PFKFB3, and PKM2 were all increased after EA intervention. More interestingly, the effects of EA would be suppressed by AMPK knockdown suggesting that EA at the DU20 and DU24 acupoints could activate AMPK to regulate glucose metabolism improving the cognitive function of APP/PS1 mice.

The APP/PS1 double transgenic mice have been extensively employed in AD research. It was previously found that the learning and memory abilities of APP/PS1 mice were impaired at the 3rd month tested by Morris water (Edwards et al., 2014). Aβ plaque is a typical pathological manifestation of AD, and Aβ deposition could be detected in APP/PS1 mice aged 4 months (Malm et al., 2011; Ramos-Rodriguez et al., 2013). Abnormal glucose metabolism is one marker of AD. There was one study longitudinally examined glucose metabolism in APP/PS1 mice at the 2nd, 3.5th, 5th, and 8th month and found that the glucose metabolism was changed throughout the progression of the disease in many brain regions, such as the entorhinal cortex, hippocampus, and frontal cortex (Li et al., 2016). In this study, 4-month-old APP/PS1 transgenic mice were selected to conduct early EA treatment. The results of the new object recognition test showed that the recognition memory of APP/PS1 mice was impaired in the 4th month.

Mitochondria are important sites for glucose metabolism where ATP synthesis occurs. However, the mitochondrial architecture is disrupted in the early stage of AD, making AG a supplement source of brain energy (Butterfield and Halliwell, 2019; Lautenschager and Kaminski, 2019). It is regrettable that the AG itself is disrupted as the disease stage progresses. It was found that the spatial distribution of the AG is consistent with the Aβ-containing plaques in the brain of preclinical patients with AD (Vlassenko et al., 2010). Autopsy studies have found that the expression of glucose transporter genes (GLUT-3), the glycolysis flux, and its key rate-limiting enzyme activity was reduced in brain regions susceptible to AD (An et al., 2018). Additionally, one study found that PDK1 was also tended to be reduced in the cerebral cortex of patients with AD which is associated with brain AG declines (Newington et al., 2012). A higher rate of glycolytic often presented in the early stages of AD, which has been considered to be a compensatory mechanism for lower glucose metabolism. However, the process of AG was also impaired with disease progression, which eventually accelerated the deposition of senile plaques and caused irreversible neuronal loss and synaptic dysfunction leading to cognitive impairment (Poisnel et al., 2012; Verclytte et al., 2016). In this study, the glucose hypometabolism phenomenon was detected in APP/PS1 mice, but the difference was not statistically significance between two groups before EA intervention. We speculated that was an early stage when the mitochondrial dysfunction was developed in APP/PS1 mice, and at that time, AG could effectively compensate for the mitochondrial dysfunction.

A systematic review and meta-analysis revealed that EA treatment has potential advantages in improving the cognitive function and life quality of patients with AD (Shao et al., 2019). An animal study has shown that EA at Baihui (DU20) acupoint could improve the spatial learning and memory abilities of AD model mice through increasing the glucose metabolism in the hippocampus (Jia et al., 2017). Another study using 18F-FDG PET has found that the glucose metabolism levels in the hippocampus and frontotemporal cortex were increased after acupuncture intervention, accompanied by the improved learning and memory abilities tested using the Y-type maze (Lai et al., 2016). Our previous 18F-FDG study also found that EA at DU20 acupoint significantly increased glucose metabolism in the cerebral cortex and hippocampus of APP/PS1 transgenic mice (Liu et al., 2017). In this study, we found that the learning and memory abilities of APP/PS1 mice were improved after EA at the DU20 and DU24 acupoints for 4 weeks. Meanwhile, the results of CEST imaging showed that the level of glucose metabolism in APP/PS1 mice was significantly increased.

As a powerful metabolic regulator, activated AMPK and its downstream proteins are extensively involved in glucose metabolism, playing a critical role in the recovery of damaged neurons (Wang et al., 2012). For its great potential to delay the onset or progression of AD, AMPK has emerged as a key target for the treatment of AD in recent years. In this study, it was found that the glucose metabolism level of APP/PS1 transgenic mice was further reduced after AMPK knockdown. With deficient mitochondrial function in AD, the process of glucose oxidative phosphorylation is hindered, resulting in neurons using AG for glucose metabolism. It was found that AG is significantly reduced in AD, while upregulation of this process could attenuate Aβ-induced neurotoxicity (Bisri et al., 2016). In certain brain regions, the increase of AG is associated with an enrichment gene expression involved in synaptic plasticity, an important neural basis for learning and memory abilities (Magistretti, 2014).

Hexokinase is the most important rate-limiting enzyme of the AG, known for four subtypes, namely, HK-I, HK-II, HK-III, and HK-IV. Although HK-I and HK-II both contain the glucose kinase domain, only the glucose kinase domain of HK-II presents activity, which is not affected by the negative feedback of glucose-6- phosphate (Tan and Miyamoto, 2015). Studies have shown that HK2 expression levels were significantly decreased in AD (Cuadrado-Tejedor et al., 2011) while enhancing HK-II activity through Wnt Signaling is capable of stimulating glycolysis rate to increase glucose uptake in cortical neurons (Cisternas et al., 2016). Another study also demonstrated that activated AMPK could upregulate the HK-II level to improve glycolysis flux contributing to increased glucose metabolism under the condition of insufficient ATP supply (Hung et al., 2017). In addition, changes in HK-II expression might be associated with the pathological manifestation in specific brain regions of AD (Rosa and Cesar, 2016). PFKFB3 is the isotype 3 of the PFKFB family, with the highest kinase and phosphatase activity among all isotypes (Sakakibara et al., 1997), which could also promote the process of AG (Bando et al., 2005). Studies have found that activated AMPK could promote the activation of PFKFB3, increasing the process of AG when the production of glycolysis was decreased (Yi et al., 2019). The PKM2, one of the four types of pyruvate kinases, is also abundant in brain tissue (Ye et al., 2012). Studies have found that there is feedback regulation between AMPK and PKM2 to promote AG (Liu et al., 2019). Our study found that EA at the DU20 and DU24 acupoints could improve the expression of AMPK, HK, PFK, and PKM2 in the hippocampus, and the beneficial effects are reversed by AMPK knockdown.

## Conclusion

Through AMPK-knockdown in APP/PS1 mice, this study demonstrated that EA treatment could activate AMPK to enhance AG of glucose metabolism in the hippocampus to improve the learning and memory abilities in APP/PS1 mice.

## Data Availability Statement

The original data supporting the conclusions of this article are available on request to the corresponding authors.

## Ethics Statement

The animal study was reviewed and approved by Fujian University of Traditional Chinese Medicine Laboratory Animal Center. Written informed consent was obtained from the owners for the participation of their animals in this study.

## Author Contributions

JT, LC, SL, and WL contributed to conception and design of the study. JL, BZ, WJ, and MY collected the data. YZ, JZ, LL, TJ, and ZW performed the statistical analysis. JL and BZ wrote the first draft of the manuscript. SL and WL revised the manuscript. All authors contributed to manuscript revision, read, and approved the submitted version.

## Conflict of Interest

The authors declare that the research was conducted in the absence of any commercial or financial relationships that could be construed as a potential conflict of interest.

## Publisher’s Note

All claims expressed in this article are solely those of the authors and do not necessarily represent those of their affiliated organizations, or those of the publisher, the editors and the reviewers. Any product that may be evaluated in this article, or claim that may be made by its manufacturer, is not guaranteed or endorsed by the publisher.

## Footnotes

1
http://www.softmaze.com

2
http://www.itksnap.org/
